# Plasma exchange combined with low-dose rituximab treatment: A case report of severe antineutrophil cytoplasmic antibody (ANCA)-associated vasculitis in the elderly

**DOI:** 10.1097/MD.0000000000044822

**Published:** 2025-09-26

**Authors:** Zhiying Wang, Xuwei He, Bokai Cheng, Zhen Wu, Qiangguo Ao

**Affiliations:** a Department of Nephrology, The Second Medical Center of Chinese PLA General Hospital, National Clinical Research Centre for Geriatric Diseases, Beijing, China.

**Keywords:** ANCA-associated vasculitis, elderly, plasma exchange, rituximab, treatment

## Abstract

**Rationale:**

Antineutrophil cytoplasmic antibody (ANCA)-associated vasculitis (AAV) is a form of necrotizing vasculitis not mediated by immune complexes, affecting multiple organs. The disease is particularly dangerous in its active stage, with high morbidity and mortality rates, especially among older patients. This study provides a possible treatment for ANCA in the elderly.

**Patient concerns:**

An 84-year-old man developed an intermittent cough of unknown etiology in early February 2024, accompanied by hemoptysis, sore throat, dry mouth, arthralgia, fatigue, and occasional low fever. He had a history of chronic kidney disease for over 14 years and had been diagnosed with AAV and ANCA-associated nephritis for more than 9 months.

**Diagnoses:**

Laboratory tests in the rheumatology department revealed C-ANCA positivity, proteinase 3 > 200 RU/mL, and an ANCA + proteinase 3 antibody concentration of 2300 IU/L. Serum creatinine concentration had increased to 457 μmol/L↑. Other autoantibodies, including anti-extra nuclear antigen, were negative. Based on these findings and the patient’s clinical symptoms, the diagnosis of ANCA-associated vasculitis was confirmed. Imaging revealed patchy and reticular opacities in both lungs, with a butterfly-wing distribution along the hila, accompanied by a small amount of bilateral pleural effusion. Scattered petechiae were observed on both upper limbs.

**Interventions:**

The patient’s condition was critical and rapidly progressing. He received successive treatments, including methylprednisolone sodium succinate + cyclophosphamide, plasma exchange, blood purification, and repeated small-dose courses of rituximab and belimumab.

**Outcomes:**

After 6 months of treatment, the patient achieved clinical, radiological, and serological remission. Hemoptysis stopped, renal function improved, immune status returned to normal, and there was no sign of ANCA recurrence. He was successfully discharged.

**Lessons:**

Rituximab offers a promising therapeutic option for older patients with chronic kidney disease complicated by AAV. Multiple small-dose rituximab regimens may reduce drug-related side effects, significantly lower treatment costs, and provide an effective, economically viable approach for clinical management.

## 1. Introduction

Antineutrophil cytoplasmic antibody (ANCA)-associated vasculitis (AAV) is a group of systemic vasculitis involving predominantly small blood vessels.^[1]^ AAV includes 3 clinical types: microscopic polyangiitis, granulomatosis with polyangiitis, and eosinophilic granulomatosis with polyangiitis. Patients with AAV have a mortality rate of up to 80% within 1 year. The 5-year mortality rate can be reduced to 25% with pharmacologic treatment.^[2]^ The disease mainly affects blood-rich organs, such as the kidneys, lungs, nervous system, skin, and gastrointestinal tract.^[3]^ In this patient, multiple large ecchymoses were present throughout the body, and scattered petechiae were found on both upper limbs (Fig. 4G and H). The natural course of AAV is progressive and fulminant, with an average survival of only 5 months. The main causes of death are vasculitis and infections, which can lead to rapid deterioration and death when combined with renal or respiratory failure.^[4]^ AAV is a rare systemic autoimmune disease. The reported incidence in Europe and North America increases with age, peaking between 60 and 70 years. The incidence and prevalence in China remain unclear. Older individuals with AAV often have declining physical function, frailty, multiple comorbidities (such as diabetes mellitus, coronary heart disease, etc), high infection risk, and poor drug tolerance, all of which increase treatment difficulty.^[5]^ Current treatment for AAV is mainly divided into the induction phase, maintenance phase, and relapse treatment.^[3]^

**Figure 1.**
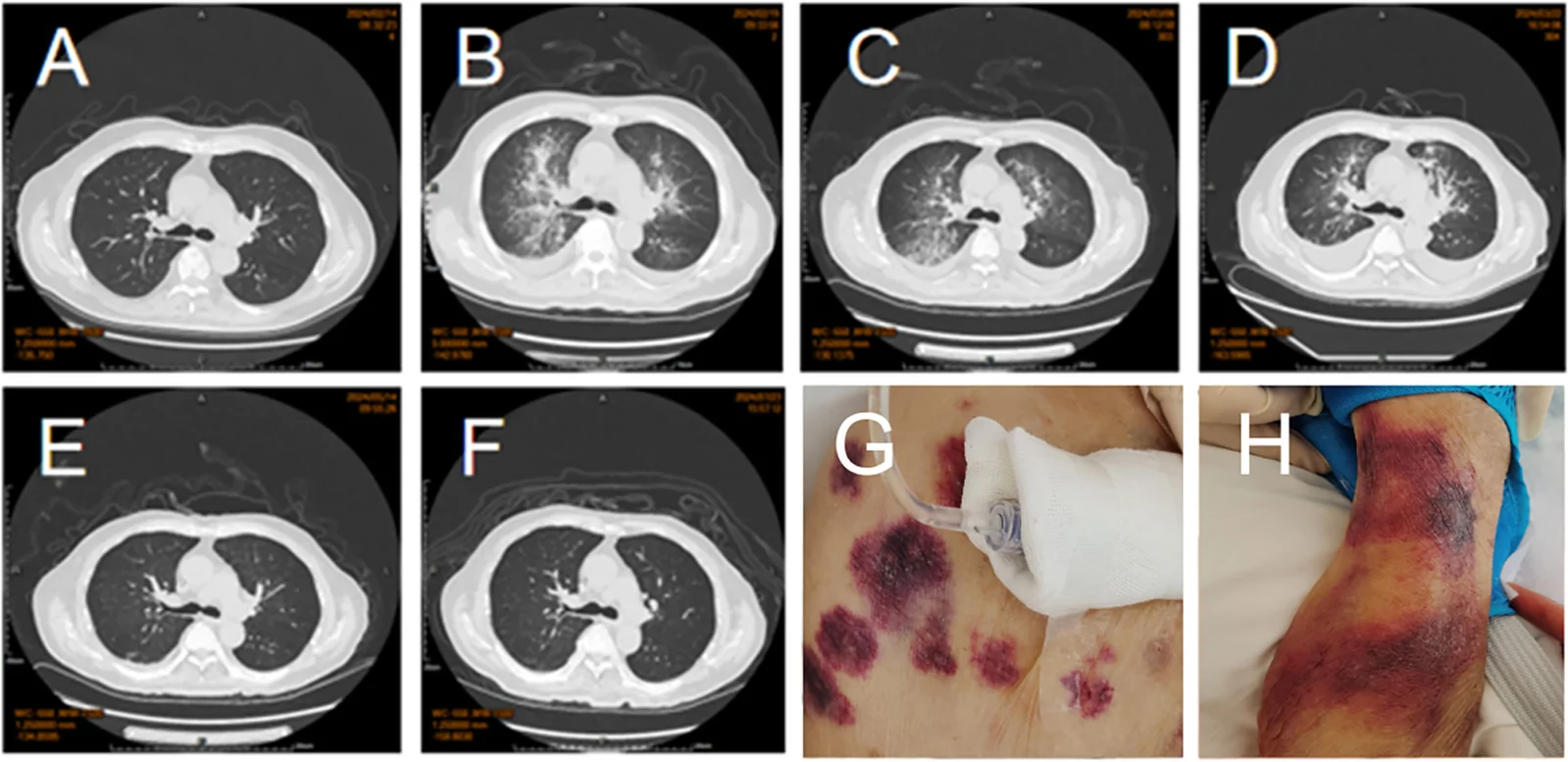
Trends in treatment regression and skin petechiae and hemorrhages on CT chest. (A) On February 14th, scattered vague patchy shadows were observed in the right upper lung and the left lower lung, suggesting pneumonia. (B) On February 19th, patchy and reticular density-increased shadows were noted in both lungs, distributed in a butterfly-wing pattern along the hilum, accompanied by a small amount of bilateral pleural effusion. (C) On March 6th, the lesions in both lungs showed improvement compared to the previous status, while the pleural effusion increased. (D) On March 22nd, both lungs demonstrated further improvement, but the pleural effusion augmented. (E) On May 4th, the patchy and reticular density-increased shadows in both lungs were absorbed, and the pleural effusion significantly decreased. (F) On June 19th, a ground-glass density faint patchy shadow was identified in the posterior segment of the left upper lung apex, considering an inflammatory lesion. (G) Multiple large ecchymoses were present throughout the body. (H) Scattered petechiae were found on double upper limbs. CT = computed tomography.

**Figure 2.**
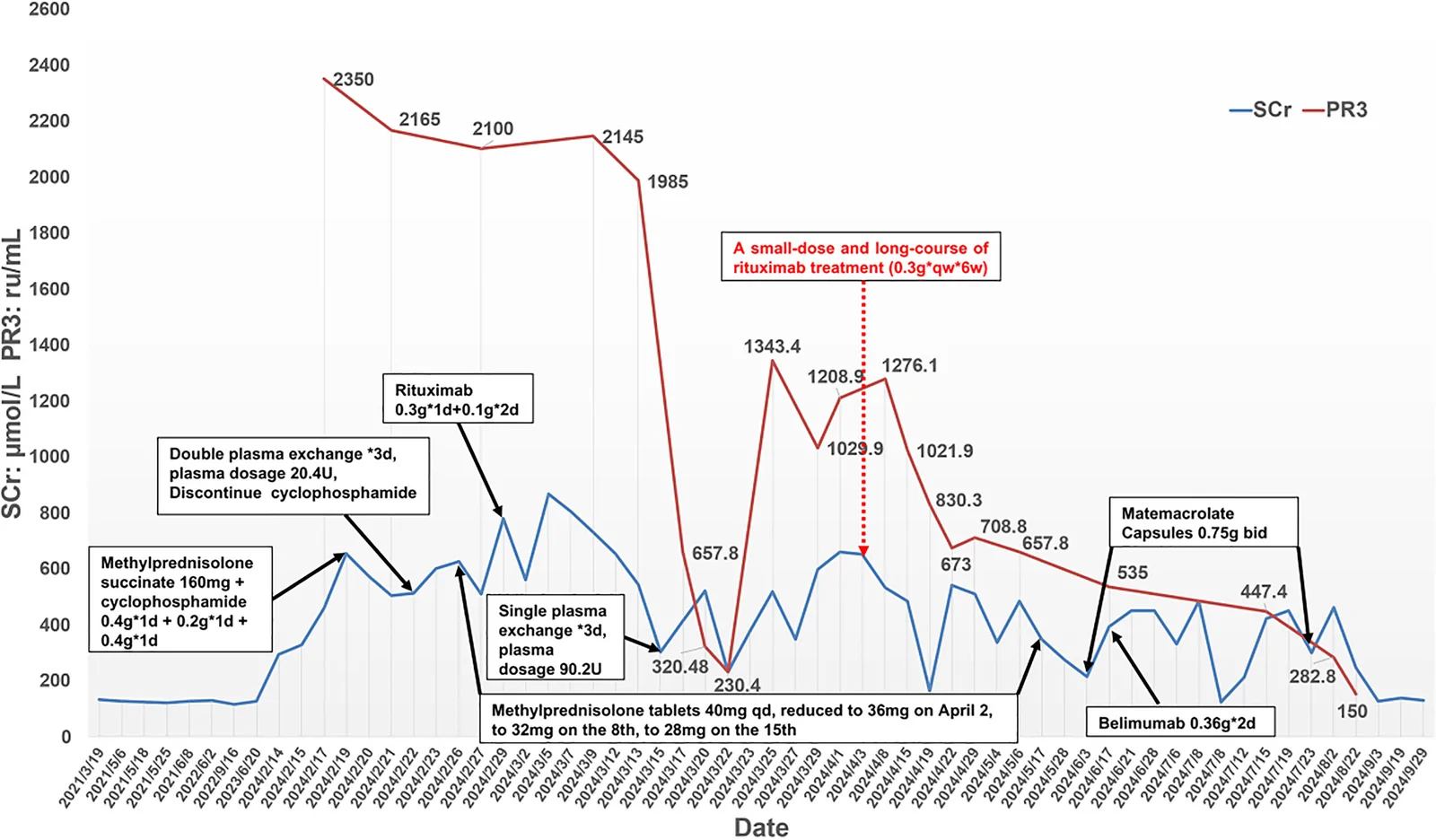
Changes in creatinine and PR3 indexes during treatment of ANCA-related vasculitis. ANCA = antineutrophil cytoplasmic antibody, SCr = serum creatinine, PR3 = proteinase 3.

**Figure 3.**
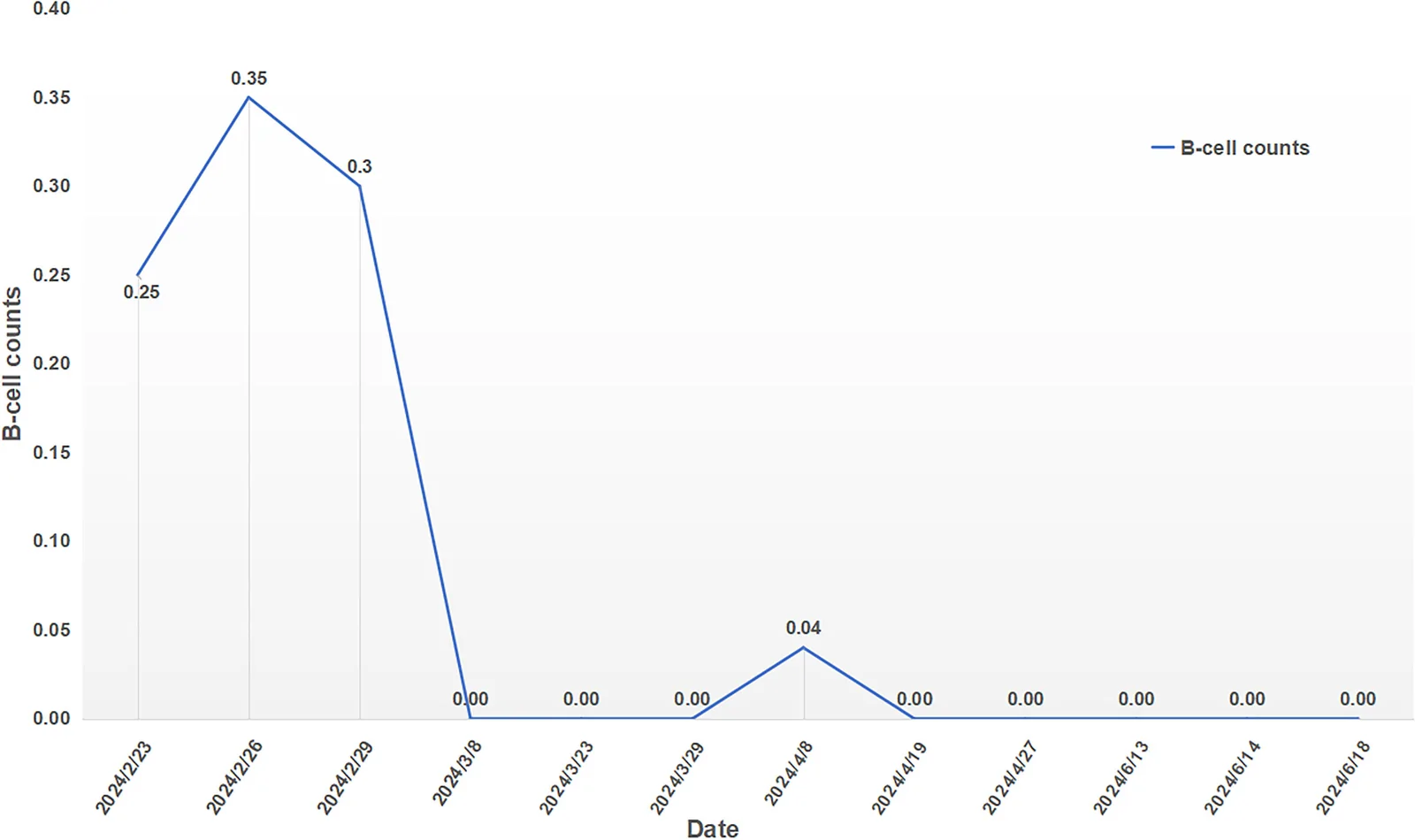
Changes in B-cell counts indexes during treatment of ANCA-related vasculitis. ANCA = antineutrophil cytoplasmic antibody.

**Figure 4.**
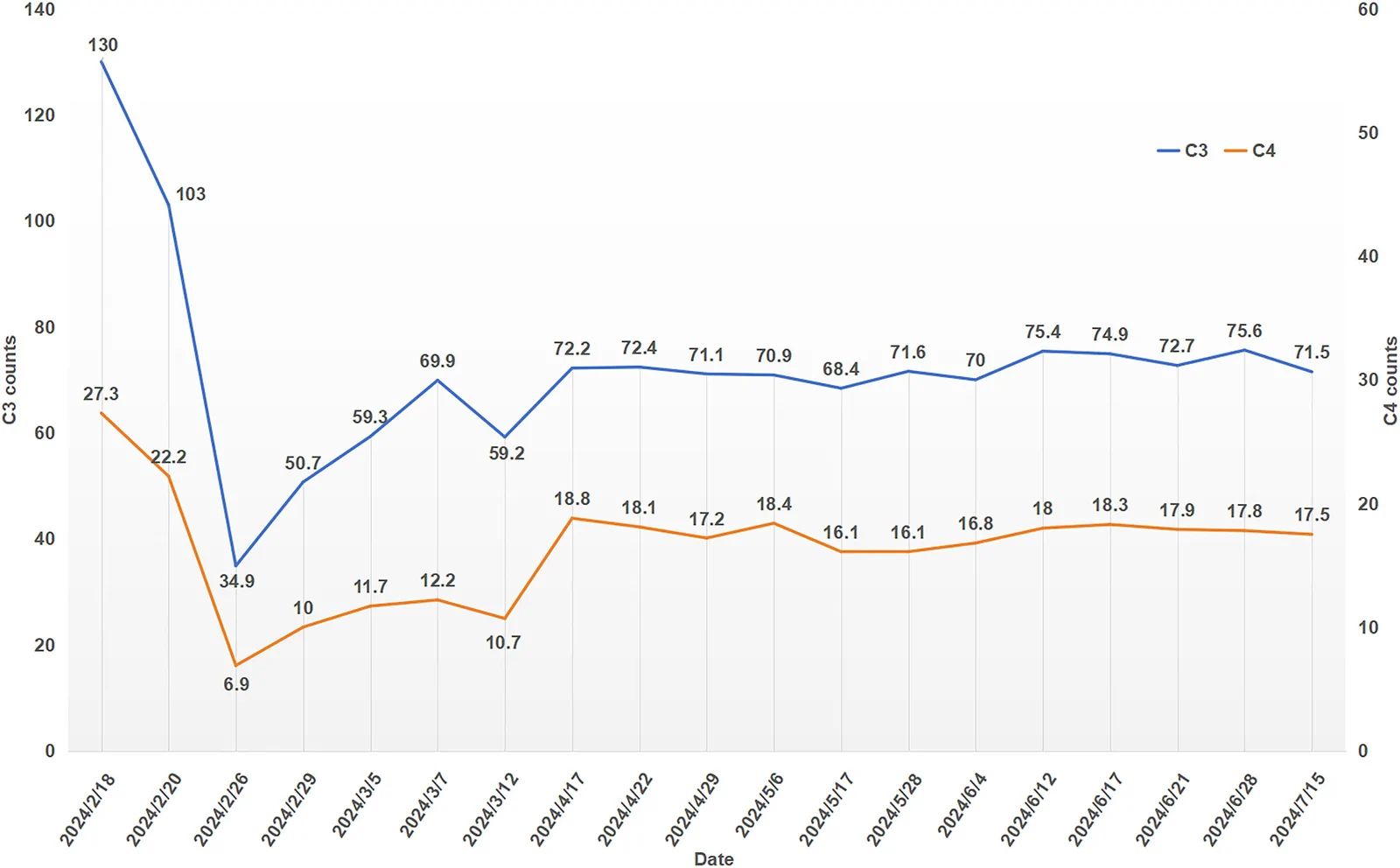
Changes in C3 & C4 counts indexes during treatment of ANCA-related vasculitis. ANCA = antineutrophil cytoplasmic antibody, C3 = complement C3, C4 = complement C4.

Rituximab (rituxan, RTX) is a human-mouse chimeric IgG1 monoclonal antibody targeting CD20, which depletes B cells to reduce ANCA production, thereby alleviating vasculitis symptoms and tissue damage.^[6]^ As a therapeutic agent for AAV, RTX demonstrated favorable efficacy and safety in a randomized trial involving 214 patients. Those receiving RTX treatment achieved complete remission following induction therapy, while RTX-based combination immunosuppression regimens showed promising results in refractory and critically ill patients.^[7–9]^ However, there are few reports on the use of rituximab in older individuals with AAV.

In this case report, we present an older patient with AAV, lung infection, and hemoptysis who experienced acute exacerbation on the background of chronic kidney disease and coronary artery disease. We adopted plasma exchange combined with multiple small-dose courses of RTX, adjusting treatment according to the patient’s changing condition over time.

## 2. Case presentation

### 2.1. Basic information

An 84-year old male patient developed intermittent cough of unknown etiology in early February 2024, with blood-streaked sputum, sore throat, dry mouth, arthralgia, fatigue, and occasional low fever. He self-administered “eucalyptus pinocchio soft capsule” and acetylcysteine effervescent tablets for symptomatic relief, but the symptoms did not improve. On the morning of February 14, he noticed dark red urine without urinary frequency, urgency, or pain, and was admitted to the emergency department of our hospital. Computed tomography (CT) of the lungs revealed scattered, fuzzy, patchy shadows in the right upper lung and left lower lung (Fig. 1A). Ultrasound of the urinary system revealed no stones or neoplasm. Blood routine: hemoglobin 120 g/L, leukocytes 9.75 × 10^9^/L, neutrophils 0.804↑, lymphocytes 0.115↓, platelets 194 × 10^9^/L; coagulation: plasma D-dimer 2.69 μg/mL↑ plasma activated partial thromboplastin time assay 61.7 seconds; biochemical tests: urea 15.6 mmol/L, creatinine 293 μmol/L↑, eGFR 19.12 mL/min/1.73 m^2^↓, potassium 4.03 mmol/L, sodium 128.0 mmol/L↓, carbon dioxide 19.4 mmol/L↓, troponin I 0.000 μg/L, and interleukin-6: 301 pg/mL↑. Urine routine: erythrocytes in full field of view, leukocytes 3 to 5/HP. He was admitted to the hospital with a provisional diagnosis of “pneumonia in both lungs, acute-on-chronic kidney disease, and hematuria (cause to be investigated).” His medical history included hyperlipidemia for 23 years (on oral atorvastatin calcium 20 mg, 1/night, with good lipid control). In May 2009, elevated serum creatinine was noted but ignored. In November 2010, he was diagnosed with chronic renal insufficiency and began treatment with Bailin capsules and other nephrology medications. Creatinine was regularly monitored and remained between 110 and 130 μmol/L. In June 2014, an oral glucose tolerance test showed a 2-hour postprandial blood glucose of 8.41 mmol/L, leading to a diagnosis of impaired glucose tolerance. Lifestyle modification and increased exercise were advised, without hypoglycemic drugs; subsequent 2-h postprandial blood glucose ranged from 8.96 to 9.29 mmol/L, glycated hemoglobin 5.3 to 5.5%. He was diagnosed with coronary artery disease in June 2017 and began long-term aspirin therapy, with no recent symptoms of chest tightness or chest pain.

### 2.2. Admission

Temperature 36.7℃, respiratory rate 22 breaths/min, heart rate 71 beats/min, blood pressure 131/71 mm Hg. He was conscious with a normal facial appearance. Respiratory movements were symmetrical, rhythmical, and tactile fremitus was equal bilaterally. Lung percussion was clear, and breath sounds were normal; no rhonchi or pleural friction run was detected. The abdomen was soft, without pressure or rebound pain, tenderness in the liver, spleen, or kidney area. There were no joint deformities, pressure pain, redness, swelling of the skin, or restriction of joint movement. There was no edema in either lower limb, and dorsal pedal pulses were normal.

### 2.3. Disease development and treatment

On February 14, scattered vague patchy shadows were observed in the right upper and the left lower lungs, suggesting pneumonia (Fig. 1A). Treatment was initiated after admission. On February 15, blood routine showed the following: hemoglobin 10^3^ g/L↓, erythrocyte 3.23 × 10^12^/L↓, leukocyte 9.84 × 10^9^/L, neutrophil 0.780↑, lymphocyte 0.1271, platelet 186 × 10^9^/L; biochemistry: urea 19.7 mmol/L↑, creatinine 328 μmol/L↑, potassium 3.87 mmol/L, sodium 127.4 mmol/L↓, carbon dioxide 20.4 mmol/L, NT-proBNP 2752.0 pg/mL↑; coagulation: prothrombin time 19.2 seconds, plasma activated partial thromboplastin time 61.7 seconds↑, plasma plasminogen time 18.2 seconds↑, plasma plasminogen activity 55%↓, international normalized ratio 1.52↑, plasma fibrinogen assay 7.40 g/L↑, plasma D-dimer 2.69 μg/mL↑, plasma antithrombin III 67%1; urinary NAG enzyme: 16.21 U/L; urinary microscopy: erythrocyte in full field. Abdominal ultrasound showed normal-sized kidneys with irregular morphology. The patient’s condition progressed rapidly, with intermittent fever peaking at 39℃. Hemoptysis became bright red, 10 to 30 mL/day. Urine output reduced compared to the volume before admission, with a full-day urine volume of 400 to 650 mL, bright red in color, without clots. Scattered petechiae and hemorrhages appeared over the whole body with a dental ulcer. On February 18, rheumatology results showed the following: C-ANCA positive, proteinase 3 (PR3) > 200 RU/mL, ANCA + PR3 antibody concentration 2300 IU/L, creatinine 457 μmol/L↑. Other autoantibodies, including anti-extranuclear antigen, were negative. Based on these findings and the clinical presentation, AAV was diagnosed, ANCA-associated nephritis was not excluded, and acute-on-chronic kidney disease was considered. At 21:40 on February 18, the patient developed orthopnea with obvious wet rales, coughing white foamy sputum with hemoptysis. Chest CT on February 19th showed marked changes compared to admission: interstitial exudative shadows in the lungs, obvious solid lesions at the pulmonary hila, and obvious bronchial dilatation (Fig. 1B). Troponin I was elevated at 0.552 μg/L↑, NT-proBNP at 69006.3 pg/mL↑. Electrocardiogram showed extensive ST depression, consistent with myocardial ischemia. Clinically, acute renal failure and acute pulmonary edema were considered. The Birmingham Vasculitis Activity Score was 33.

This older patient had a clear diagnosis of AAV, presenting with symptoms of pulmonary infection, hemoptysis, hematuria, and skin ecchymosis. On top of preexisting chronic kidney disease and coronary heart disease, the condition acutely worsened, making treatment challenging, complex, and associated with a high mortality risk. Based on disease progression at different stages, a 4-phase treatment approach was implemented.

Phase 1: on February 14, anti-infection treatment was initiated with intravenous infusion of cefoperazone sodium and tazobactam sodium 2.25 g twice daily. A chest CT on February 19 revealed patchy and reticular opacities with increased density in both lungs. As the infection was not controlled, the antibiotic regimen was adjusted to meropenem (Sumitomo-JP) 0.5 g every 8 hours (discontinued on March 7 after 18 days of cumulative use) plus caspofungin acetate 50 mg once daily (discontinued on April 2 after 44 days of cumulative use). On the same day, blood biochemical tests showed elevated creatinine at 653 μmol/L↑ and urea at 33.2 mmol/L↑. Following multidisciplinary consultation and comprehensive evaluation of the patient’s indicators, continuous renal replacement therapy (CRRT) was initiated at 8 hours per day with a replacement fluid volume of 32 L. Concurrently, intravenous methylprednisolone sodium succinate 160 mg plus cyclophosphamide (CYC) 0.4 g × 1 d + 0.2 g × 1 d + 0.4 g × 1 d for 3 consecutive days was administered. Hemoptysis persisted, and ANCA levels showed no declining trend. On February 22, the patient was found to have trilineage cytopenia: red blood cells 2.66 × 10^12^/L↓, white blood cells 5.86 × 10^9^/L, and platelets 66 × 10^9^/L↓. Due to poor therapeutic response to CYC and uncontrolled disease progression, CYC treatment was discontinued.

Phase 2: from February 22 to 24 (3 consecutive days), the patient underwent 2-hour daily double filtration plasmapheresis, with a total replacement volume of 20.4 units of type B Rh(D)-positive leukocyte-depleted plasma. Concurrently, 80 mg of methylprednisolone sodium succinate was administered intravenously. On February 26, oral glucocorticoids (GCs) were initiated, with methylprednisolone tablets tapered gradually from 40 mg to 8 mg (Table 1). However, therapeutic efficacy remained unsatisfactory as hemoptysis persisted and PR3 levels showed no decline.

**Table 1:** Usage of glucocorticoids (methylprednisolone).

Start time	Stop time	Weeks	Dosage (mg)	Remarks
February 26, 2024	March 18, 2024	3	40	The 3rd wk
March 18, 2024	April 1, 2024	2	36	The 5th wk
April 1, 2024	April 7, 2024	1	32	The 6th wk
April 7, 2024	April 15, 2024	1	28	The 7th wk
April 15, 2024	May 6, 2024	3	24	The 10th wk
May 6, 2024	May 27, 2024	3	20	The 13th wk
May 27, 2024	June 17, 2024	3	16	The 16th wk
June 17, 2024	July 8, 2024	3	12	The 19th wk
July 8, 2024	July 29, 2024	3	10	The 22nd wk
July 29, 2024	August 19, 2024	3	6	The 25th wk
August 19, 2024	September 9, 2024	3	4	The 28th wk
September 9, 2024	Up till now		4	

Phase 3: on March 1, the patient’s white blood cell count dropped from 9.38 × 10^9^/L on February 19 to 2.11 × 10^9^/L, raising concerns about the risk of severe infection. Although the PR3 concentration showed a slight decrease, hemoptysis persisted. Vasculitis showed no significant improvement, infection remained uncontrolled, and renal function exhibited no notable recovery. Consequently, an individualized treatment plan was implemented for this older patient: low-dose RTX induction therapy. On March 1, 2, and 3, RTX was administered at 0.3 g × 1 d + 0.1 g × 2 d, totaling 0.5 g over 3 days. After 1 month of discontinuation, the PR3 antibody concentration declined markedly (Fig. 2). From March 8 onward, the B lymphocyte count remained at 0. During this period, CRRT was administered every other day for 8 hours (32 L replacement fluid volume). On March 19, 20, and 21, single plasmapheresis (Plasma Exchange PE) was performed, cumulatively exchanging 90.2 U of type B Rh(D)-positive leukocyte-reduced plasma over 3 days. Although the PR3 antibody concentration dropped to a low value of 230.4 RU/mL, it could not be sustained through plasmapheresis alone.

On April 3, a low-dose, long-course RTX regimen was initiated: 0.3 g per dose, once weekly for 6 weeks (total cumulative RTX dose: 1.8 g). To mitigate drug side effects, control cumulative damage, prevent relapse, and manage comorbidities (particularly infection, cardiovascular disease, and malignancy), vital signs were closely monitored during treatment. Regular assessments included B-cell subsets and counts (Fig. 3), C3 & C4 counts (Fig. 4), white blood cells, platelets, infection markers, cardiac enzymes, and liver and kidney function. Complement and PR3 antibody concentrations were rechecked every other day. Additional measures included immunosuppression, anticoagulation, gastric protection, calcium supplementation, dietary regulation, and enhanced protective isolation. Daily room disinfection was performed for 1 hour using an air purifier (Honeywell, Nanjing, Jiangsu, China), and strict personal hygiene measures were enforced, including mask-wearing during out-of-room examinations to prevent infection.

This treatment regimen yielded significant results: PR3 antibody concentration continued to decline, scattered ecchymosis gradually resolved, hemoptysis ceased, gross hematuria disappeared, and chest CT showed improvement in damaged lung areas. The condition did not progress to respiratory failure requiring mechanical ventilation. Despite biweekly CRRT sessions, the patient’s pre-dialysis creatinine levels exhibited a clear downward trend, with both pulmonary and renal function showing recovery.

Phase 4: considering the rituximab dosage had been reached, mycophenolate mofetil capsules (Roche-CH) 750 mg orally twice daily + compound sulfamethoxazole tablets 0.96 g orally once nightly were administered on May 23 for infection prophylaxis. The Vasculitis Damage Index was assessed, and belimumab totaling 720 mg was administered on June 14 and June 18. ANCA concentration was evaluated on August 22 at 150 IU/L. The condition showed further remission, with a Birmingham Vasculitis Activity Score of 2 and a Vasculitis Damage Index score of 0. Pre-dialysis creatinine levels remained stable at 199 to 219 μmol/L, with urine volume maintained at 1200 to 1500 mL, allowing discontinuation of hemostasis hemodialysis. Regular follow-ups were continued to monitor complete blood count, PR3 antibody concentration, infection markers, and other indicators to prevent relapse.

## 3. Discussion

In this case, the management of this older patient with AAV involved preventing disease progression, reducing organ damage, and achieving more effective and faster remission with higher remission rates. At the same time, preventing disease development, reducing organ damage, and improving renal prognosis were of vital importance. The patient exhibited significant adverse reactions during the first phase of the conventional treatment regimen combining GC with CTX, including myelosuppression,^[10]^ which rendered the treatment ineffective. Literature reports that infections are most likely to occur in the first 3 months after RTX therapy (incidence of 30%),^[11]^ hypogammaglobulinemia (around 16%),^[12]^ and infusion-related adverse reactions (41%).^[13]^

Throughout the treatment course, the individualized regimen utilizing low-dose, long-term RTX demonstrated remarkable efficacy. Timely clinical parameter monitoring, along with appropriate protective isolation measures, such as single-room therapy and hand hygiene, proved pivotal in preventing complications, such as infection and drug-related infusion reactions. Regarding the treatment of this patient, a renal biopsy was not performed, which was originally planned to obtain histopathological results of ANCA-associated vasculitis through renal puncture biopsy to more accurately guide treatment and evaluate prognosis. However, after thorough communication with the patient and their family about the condition, although they agreed to the treatment plan, they did not consent to the renal biopsy examination due to personal considerations.

Regarding this patient’s use of oral methylprednisolone tablets, guideline recommendations indicate that a glucocorticoid tapering regimen should be implemented during the first 6 months of treatment for patients with AAV. For severe AAV cases, this tapering approach can significantly reduce the risk of serious infections (-5.9% to -12.8%) without increasing 1-year mortality risk (mortality risk reduction of -2.1%).^[14]^ Both the PEXIVAS and LoVAS studies suggest that RTX-based treatment protocols lower accumulated GC doses while reducing the incidence of severe adverse events.^[15,16]^

Considering the patient’s advanced age, severe renal impairment, and poor tolerance to glucocorticoid adverse reactions (typical in older patients) we did not employ the standard GC tapering regimen. Instead, a more cautious, individualized adjustment was implemented for both the initial glucocorticoid dosage and tapering schedule: the hormone dose was appropriately reduced, and tapering proceeded at a slower pace. This modification reduced the standard protocol’s initial methylprednisolone dosage during the first 2 weeks (patient weight 69.2 kg; initial dose reduced from 60–40 mg).

In patients with AAV, plasmapheresis (PLEX) should be considered for those requiring dialysis and/or experiencing rapid creatinine elevation (serum creatinine > 300 μmol/L), as well as extensive alveolar hemorrhage with hypoxemia. PLEX has shown no significant correlation with major outcome risks, mortality, complete remission, or adverse events. Regarding secondary outcomes, PLEX may be effective in suppressing end-stage renal disease during early treatment.^[17]^ However, research on the efficacy and safety of PLEX in older patients is limited, necessitating further clinical studies in this population. The long-term benefits of PLEX remain controversial; while it may reduce the probability of end-stage renal disease within 1 year in patients with AAV, it can also increase the incidence of severe infections.^[18]^

Due to increased vascular permeability from AAV-related vascular wall inflammation, as well as heparin use and coagulation factor loss during hemodialysis and PLEX, this patient (presenting with hemoptysis, hematuria, extensive skin ecchymosis, and hemorrhagic spots) was treated with heparin-free or low-dose, low-molecular-weight heparin protocols during PLEX or hemodialysis. While prioritizing therapeutic efficacy, close attention was paid to coagulation function, and invasive procedures were performed cautiously. Although PLEX played an adjunctive role in treatment, its potential adverse effects should not be overlooked, and strict aseptic techniques must be maintained to prevent infections.

Although the patient has currently achieved remission, the next goal is to prevent relapse. Despite ongoing maintenance therapy, more than 50% of patients with AAV still experience relapse during the disease course.^[19]^ After discharge, establishing effective communication channels with the patient and their family, providing psychological support, conducting regular phone and outpatient follow-ups, and performing reexaminations and disease assessments are crucial for improving patient adherence to the treatment regimen.

## 4. Conclusion

Plasmapheresis combined with low-dose RTX provides a promising treatment option for older patients with chronic kidney disease complicated by AAV. The use of small doses of multiple RTX administrations is likely more desirable, as it can reduce drug-induced side effects, significantly lower patients’ economic burden, and offer a valuable approach for clinical treatment. However, for older patients, clinical applications must be approached cautiously, with close attention to the effects of cyclophosphamide on the hematopoietic system. In patients with severe ANCA, dual application is unsatisfactory, while single application may be better, though it cannot completely eliminate PR3. Treatment should be comprehensively evaluated based on the specific conditions of older patients. More case studies are needed to determine the optimal dose and regimen of RTX for treating older patients with AAV. This study is an exploration of a single patient individualized treatment protocol, which still needs to be determined by a large RCT study to determine the universal treatment pattern.

## Acknowledgments

The authors thank the patient for granting permission to publish this manuscript.

## Author contributions

**Conceptualization:** Zhiying Wang, Xuwei He, Qiangguo Ao.

**Data curation:** Zhiying Wang, Xuwei He, Qiangguo Ao.

**Formal analysis:** Zhiying Wang, Xuwei He, Qiangguo Ao.

**Funding acquisition:** Qiangguo Ao.

**Investigation:** Zhiying Wang, Xuwei He, Bokai Cheng, Zhen Wu, Qiangguo Ao.

**Methodology:** Zhiying Wang, Xuwei He.

**Resources:** Bokai Cheng, Zhen Wu, Qiangguo Ao.

**Project administration:** Qiangguo Ao.

**Supervision:** Qiangguo Ao.

**Validation:** Zhiying Wang, Xuwei He.

**Visualization:** Zhiying Wang, Xuwei He.

**Writing – original draft:** Zhiying Wang, Xuwei He.

**Writing – review & editing:** Qiangguo Ao.
